# p66^Shc^ Inactivation Modifies RNS Production, Regulates Sirt3 Activity, and Improves Mitochondrial Homeostasis, Delaying the Aging Process in Mouse Brain

**DOI:** 10.1155/2018/8561892

**Published:** 2018-03-12

**Authors:** Hernán Pérez, Paola Vanesa Finocchietto, Yael Alippe, Inés Rebagliati, María Eugenia Elguero, Nerina Villalba, Juan José Poderoso, María Cecilia Carreras

**Affiliations:** ^1^Laboratory of Oxygen Metabolism, INIGEM-UBA-CONICET, Buenos Aires, Argentina; ^2^Departamento de Medicina, Facultad de Medicina, Universidad de Buenos Aires, Buenos Aires, Argentina; ^3^Departamento de Bioquímica Clínica, Facultad de Farmacia y Bioquímica, Universidad de Buenos Aires, Buenos Aires, Argentina

## Abstract

Programmed and damage aging theories have traditionally been conceived as stand-alone schools of thought. However, the p66^Shc^ adaptor protein has demonstrated that aging-regulating genes and reactive oxygen species (ROS) are closely interconnected, since its absence modifies metabolic homeostasis by providing oxidative stress resistance and promoting longevity. p66^Shc(−/−)^ mice are a unique opportunity to further comprehend the bidirectional relationship between redox homeostasis and the imbalance of mitochondrial biogenesis and dynamics during aging. This study shows that brain mitochondria of p66^Shc(−/−)^ aged mice exhibit a reduced alteration of redox balance with a decrease in both ROS generation and its detoxification activity. We also demonstrate a strong link between reactive nitrogen species (RNS) and mitochondrial function, morphology, and biogenesis, where low levels of ONOO^−^ formation present in aged p66^Shc(−/−)^ mouse brain prevent protein nitration, delaying the loss of biological functions characteristic of the aging process. Sirt3 modulates age-associated mitochondrial biology and function via lysine deacetylation of target proteins, and we show that its regulation depends on its nitration status and is benefited by the improved NAD^+^/NADH ratio in aged p66^Shc(−/−)^ brain mitochondria. Low levels of protein nitration and acetylation could cause the metabolic homeostasis maintenance observed during aging in this group, thus increasing its lifespan.

## 1. Introduction

Aging is a multifactorial degenerative process that strongly impacts the endocrinology and biochemistry of the brain [1]. Mitochondrial function declines in the normal aging process and increases the incidence of age-related disorders. In accordance with the free radical theory of aging, this process can be ascribed to the oxidative damage caused by the generation of reactive oxygen species (ROS) derived from mitochondrial respiration when O_2_ is partially reduced [2]. It has been demonstrated that electron transfer leakage throughout the mitochondrial respiratory chain is responsible for the formation of most cellular ROS [3]. In this way, ROS generation depends on mitochondrial activity [4], which by itself can be perceived as a signal sensor and transducer in various enzymatic and gene-mediated physiological and pathophysiological processes [5].

In addition, mitochondrial function and morphology are also modulated by nitric oxide (NO) exposure. NO is a relatively low reactive radical whose derivatives, such as peroxynitrite (ONOO^−^), can cause nitrosative damage in biomolecules, proteosomic degradation failure, enzymatic activity inhibition, and overall interference of regulatory functions [6]. These processes occur in aging, when the levels of ROS rise and superoxide anion (O_2_^−^) conjugates with NO to create reactive nitrogen species (RNS), and their products nitrate (NO_3_^−^), nitrite (NO_2_^−^), and peroxynitrite (ONOO^−^), which have demonstrated a direct role in cellular signaling, vasodilation, immune response, and aging [7–9].

Alterations in ROS/RNS levels have been mechanistically linked with changes in mitochondrial morphology in many studies, suggesting a crosstalk between redox homeostasis and mitochondrial dynamics [10]. Mitochondrial morphology is regulated by continuous fusion and fission events that are essential to maintaining normal mitochondrial function [11]. These fusion/fission processes are finely regulated by the mitochondrial fusion proteins optic atrophy 1 (Opa1), mitofusins 1 and 2 (Mfn1 and Mfn2, resp.) and by the mitochondrial fission proteins dynamin-related protein 1 (Drp1) and fission protein 1 (Fis1). High levels of ROS and RNS, such as those measured in aging models, have been associated with redox-induced posttranslational modifications (i.e., nitration or S-nitrosylation) in several of these proteins and their binding targets, leading to mitochondrial fragmentation [12–14].

Mitochondrial plasticity and density decrease with age, and their capacity for mitochondrial biogenesis is reduced owing to a redox-dependent decline of (peroxisome proliferator-activated receptor *γ*-coactivator-1*α*) (PGC-1*α*) activity [15–17]. PGC-1*α* is a transcriptional coactivator that enhances the activity of specific transcription factors, in turn coordinating the expression of key nuclear-encoded mitochondrial genes that are required for the proper functioning of the organelle.

A link between mitochondrial biogenesis and mitochondrial morphology has been suggested by the fact that PGC-1*α* regulates Mfn2 expression. Repression of Mfn2 in cells decreases oxygen consumption, mitochondrial membrane potential, and the expression of oxidative phosphorylation proteins [17].

On the other hand, changes observed in the lysine acetylation profile of proteins or “acetylome” are related to normal aging and age-related diseases [18]. Sirt3, a member of the NAD^+^-dependent deacetylase family, is the most studied mitochondrial sirtuin associated with metabolic homeostasis maintenance. Sirt3 is considered a key element to delaying the loss of biological functions during aging [19]. Recent studies have shown that the acetylation levels of mouse mitochondrial proteins are modulated in fasting conditions or during caloric restriction [20]. Additionally, an increase in Sirt3 expression was observed in several tissues under these conditions, suggesting an improvement in the protection against ROS-induced aging [21–24]. SIRT3 stimulates proteins participating in ATP generation, tricarboxylic acid (TCA) cycle, and electron transport chain and stress response [25, 26]. Further, it promotes mitochondrial biogenesis via PGC-1*α*-mediated inhibition of cellular ROS and delays mitochondrial dynamic age imbalance, deacetylating Opa1 [27, 28].

In the last years, more cases of cooperation between ROS and aging-regulating genes have been established in senescence and aging development [29]. The adaptor protein p66^Shc^ is a genetic determinant of lifespan that regulates ROS metabolism and cellular apoptosis. Faced with a stress signal (growth factor, insulin receptor activation, excessive radiation, or oxidative stress), PKC*β* phosphorylates p66^Shc^ in Ser36, which translocates to mitochondria, oxidizes cytochrome c in the intermembrane space, and prevents electron transfer to cytochrome oxidase, resulting in a higher reduction level of complex III (ubiquinol and cytochromes b-c1) [30]. The inhibition of electron free flow determines the formation of semiubiquinone and the subsequent O_2_ monoelectric reduction with O_2_^−^ formation, eventually dismutated to hydrogen peroxide (H_2_O_2_) by MnSOD [31]. p66^Shc^ ablation in mice is translated into a significant decrease in mitochondria-produced ROS and a 30% increase in lifespan [32]. These knockout mice for p66^Shc^ (p66^Shc(−/−)^) have been shown to be thinner, to exhibit an increased metabolic rate, and to have less body fat than their wild-type littermates [33]. And more remarkably, they have been described as an animal model of healthy aging with better cognitive abilities at adulthood in a spatial memory task and improved physical performance at senescence [34]. The aim of this study is to deepen the bidirectional relationship between redox homeostasis and the imbalance of mitochondrial dynamics and biogenesis in this model of healthy aging.

## 2. Materials and Methods

### 2.1. Mice and Ethics

Dr. P. G. Pellicci at the European Institute of Oncology, Milan, kindly provided 129 p66^Shc^ knockout (p66^Shc(−/−)^) breeding pairs for this project. The breeding stocks were backcrossed onto 129/SV mice. A colony was maintained in the animal facility at the School of Pharmacy and Biochemistry of the University of Buenos Aires. Heterozygous Shc KO mice (p66^Shc(+/−)^) were mated to produce the homozygous Shc KO (p66^Shc(−/−)^), and WT control littermates were used for the study. Mice aged 3, 18, and 24 months from both groups were housed in a constant-temperature room (20–24°C) with a 12-hour dark/12-hour light cycle, fed ad libitum with a standard diet and free access to water. Animal experiments were performed in accordance with the principles of laboratory animal care. The Institutional Animal Care and Research Committee of the University of Buenos Aires approved all animal procedures. We made every possible effort to minimize animal suffering and to reduce the number of animals used.

### 2.2. Mitochondrial Isolation

Mice were deeply anesthetized using ketamine (50 mg/kg) and sacrificed by cervical dislocation. The brains were immediately extracted and homogenized in MSHE buffer (0.22 M mannitol, 70 mM sucrose, 0.5 mM EGTA, 2 mM KHEPES). The homogenate was centrifuged at 800*g* for 10 min at 4°C. The supernatant was transferred and centrifuged again at 10,000*g* for 10 min at 4°C in a Sorvall centrifuge. The pellet containing mitochondria was resuspended and isolated at 100,000*g* by Percoll gradient centrifugation in an ultracentrifuge [35]. Fresh mitochondria were used to determine membrane potential, oxygen consumption, NO production, nNOS activity, and NAD/NADH ratio. For complex activities, MnSOD and catalase activity, and Western blotting determination, mitochondria were stored at −80°C and then subjected to three freeze/thaw cycles followed by a homogenization step by passage through a 29 G hypodermic needle.

### 2.3. NOS Activity

Homogenate fractions of brain tissue from control and KO (p66^Shc−/−^) mice were determined by conversion from [3H] L-Arg to [3H] L-citrulline [36] in 50 mM potassium phosphate buffer, pH 7.4, in the presence of 100 *μ*M L-Arg, 0.1 *μ*M [3H] L-Arg (NEN), 0.1 mM NADPH, 0.3 mM CaCl_2_, 0.1 *μ*M calmodulin, 10 *μ*M tetrahydrobiopterin, 1 *μ*M flavin adenine dinucleotide, 1 *μ*M flavin mononucleotide, 50 mM L-valine, and 1 mg/mL protein. Specific activity was calculated by subtracting the remaining activity in the presence of the NOS inhibitor 5 mM L-NMMA or 2 mM ethylene glycol tetraacetic acid [37].

### 2.4. Mitochondrial H_2_O_2_ Production

Mitochondrial H_2_O_2_ production was monitored using a Hitachi F-2000 spectrofluorometer with excitation and emission wavelengths at 315 and 425 nm, respectively [31]. The reaction medium, potassium phosphate buffer (50 mM) and 50 mM L-valine, was supplemented with 10 mM succinate, 12.5 units/mL horseradish peroxidase, 250 M *p*-hydroxyphenylacetic acid, and 0.15 mg of mitochondrial protein per mL. Mitochondrial preparations were supplemented with 1 *μ*M Mn(III)tetrakis(4-benzoic acid) porphyrin (Cayman Chemical, Ann Arbor, MI) and 0.2 *μ*M antimycin A for a uniform maximal H_2_O_2_ production rate [38].

### 2.5. Antioxidant Enzyme Activities

Mitochondrial Mn-SOD activity was determined by inhibition of cytochrome c reduction at 550 nm in 50 mM potassium phosphate buffer/0.1 mM EDTA (pH 7.8) at 25°C, and results were expressed as pmoles SOD per mg of protein [39]. Catalase activity in supernatants was determined by the decrease in H_2_O_2_ absorption at 240 nm (*ε*_240_ = 41 *μ*M^−1^ cm^−1^). The pseudo-first reaction constant (*k*′) expressed as *k*′/mg protein was calculated and then extrapolated to a calibration curve to obtain U CAT/mg protein [40].

### 2.6. Oxygen Consumption Rate

Oxygen uptake was determined polarographically with a Clark-type electrode placed in a 3 mL chamber at 30°C, in a reaction medium consisting of 0.23 mM mannitol, 70 mM sucrose, 30 mM Tris-HCl, 4 mM MgCl_2_, 5 mM Na_2_HPO_4_-KH_2_PO_4_, and 1 mM EDTA, pH 7.4, saturated with room air (225 *μ*M O_2_) with 0.5–1 mg mitochondria protein/mL. Oxygen uptake was determined using 6 mM malate-glutamate or succinate as substrates in the presence (state 3) or the absence (state 4) of a phosphate acceptor (0.2 mM ADP). Oxygen uptake was expressed in nanogram atoms of oxygen per minute per milligram of protein. The respiratory control rate was calculated as state 3/state 4 respiration rate. The P/O ratio was calculated as the ratio of nmoles of added ADP per nanogram atoms of O_2_ utilized during state 3 [41, 42].

### 2.7. Mitochondrial Respiratory Chain Complex Activity

Nicotinamide adenine dinucleotide- and succinate-cytochrome c reductase activities (complexes I–III and II–III, resp.) were assayed by cytochrome c reduction at 550 nm with a Hitachi U3000 spectrophotometer (Hitachi, Tokyo, Japan) at 30°C. Cytochrome oxidase activity (complex IV) was determined by monitoring cytochrome c oxidation at 550 nm (*ε*_550_ = 21 mM^−1^ × cm^−1^); the reaction rate was measured as the pseudo-first-order reaction constant (*k*′) and expressed as *k*′/min/mg protein [43].

### 2.8. Mitochondrial Membrane Potential (Ψ) and Mitochondrial NO, ROS, and O_2_^−^ Production

Mitochondrial membrane potential (Ψ) and mitochondrial NO, ROS, and O_2_^−^ production were estimated by flow cytometry (FACSCalibur) using fluorescent dyes 3,3′-dihexyloxacarbocyanine iodide (DiOC6) (200 nM; Molecular Probes), DAF-FM (10 *μ*M; Molecular Probes) with 0.3 mM L-arginine in the presence and absence of the NOS inhibitor (3 mM L-NAME), H_2_DCFDA (25 *μ*M; Sigma-Aldrich), or MitoSOX (2.5 *μ*M; Molecular Probes), respectively. Fresh brain mitochondria (50 *μ*g protein/mL) were suspended in 0.5 mL of respiration buffer (120 mM KCl, 5 mM KH_2_PO_4_, 1 mM EGTA, 3 mM HEPES, and 1 mg/mL fatty acid-free BSA [pH 7.4]). These assays were performed in the dark at 37°C for 15 min. After the incubation period, mitochondria were centrifuged for 5 min at 10,000*g* for pelleting and washing and then suspended to record the fluorescence intensity.

### 2.9. NAD/NADH Mitochondrial Ratio and ATP Content

The NAD Cycling Enzyme Mix in Abcam NADH/NAD Quantification Kit was specifically used to recognize NADH/NAD^+^ in an enzyme cycling colorimetric reaction read at OD 450 nm in a colorimetric microplate reader. Total ATP content was determined in 10 mg of fresh slice brain tissue based on luciferase's requirement for ATP in producing light (emission maximum ~560 nm at pH 7.8) using the ATP bioluminescent assay kit (Molecular Probes).

### 2.10. SIRT3 Deacetylase Activity

SIRT3 deacetylase activity was determined as described in Cayman's SIRT3 direct fluorescent screening assay kit (item no. 10011566). In a plate, assay buffer, modified p53 acetylated peptide, recombinant Sirt3, and NAD^+^ were incubated with increased concentrations of ONOO^−^ (100, 250, and 500 *μ*M) or with purified mitochondrial lysates, final volume 50 *μ*L. Deacetylation reactions were stopped after 2 h of incubation at room temperature by adding 50 *μ*L of stop solution. The fluorophore release by deacetylation of the p53 peptide was analyzed with a fluorometer using an excitation wavelength of 350–360 nm and an emission wavelength of 450–465 nm [44].

### 2.11. Mitochondrial Morphology

Transmission electron microscopy was performed in 90 nm sections from mouse brains and fixed in 4% paraformaldehyde, 2% glutaraldehyde, and 5% sucrose in PBS, followed by 2 h postfixation in 1% osmium tetroxide and then 1 h in uranyl acetate in 50% ethanol. Samples were washed with 50% ethanol and dehydrated with a graded series of ethanol, clarified with acetone, and embedded in Vestopal. Grids were prepared and stained with uranyl acetate and lead citrate. Samples were observed at 100 kV with a Zeiss EM 109T transmission electron microscope (Zeiss, Oberkochen, Germany). The mitochondrial area and length were measured in 400 mitochondria per mouse using the ImageJ software. For the quantification of mitochondrial morphology, we used the criteria described in [45].

### 2.12. RNA Extraction, Quantitative Real-Time PCR, and mtDNA Content

Total RNA was extracted with TRIzol Reagent (Invitrogen Corp.). After DNase treatment, 2 *μ*g of samples was reverse-transcribed in duplicate using *Taq* polymerase and Oligo d(T)_16_. For RT-qPCR studies, cDNA samples were diluted 5-fold, and for mtDNA/nDNA qPCRs, 40 ng of total genomic DNA was used. PCR amplification and analyses were performed with StepOnePlus Real-Time PCR Systems (Thermo Fisher). SYBR® Green PCR Master Mix (Thermo Fisher) was used for all reactions, following the manufacturer's instructions. Total DNA was precipitated with isopropanol (vol. 1 : 1) from brain tissue samples (∼10 mg), homogenized in 20 mg/mL of proteinase K solution, and finally dissolved in Tris-EDTA buffer. The mtDNA/nDNA ratio was calculated using mtDNA primers for the 16S rRNA and nDNA primers for the *β*-2 microglobulin (*β*2M) gene [46].

### 2.13. Western Blots

15 to 70 *μ*g of protein was electrophoresed on 10–15% vol/vol polyacrylamide SDS-PAGE gels. Proteins were electrophoretically transferred onto PVDF membranes. The membranes were subsequently blocked and, after blocking, were incubated at room temperature with the following antibodies: 1 : 500 anti-NOS1 (R-20) : sc-648, 1 : 500 anti-Mfn2 (H-68) : sc-50331, 1 : 2000 anti-actin (I-19) : sc-1616, and 1 : 2000 anti-VDAC1 (N-18) : sc-8828 were obtained from Santa Cruz, CA. 1 : 500 anti-OPA1 : 612607 and 1 : 1000 anti-DLP1 : 611113 were obtained from BD Biosciences. 1 : 1000 anti-phospho-DRP1 (Ser616) : #3455 was obtained from Cell Signaling. 1 : 1000 anti-nitrotyrosine, clone1A6 : #05-233 was obtained from EMD Millipore.

### 2.14. Coimmunoprecipitation

Mitochondrial proteins (500 *μ*g) were incubated with 4 *μ*g of monoclonal anti-Sirt3 antibody (C73E3) and 30 *μ*L protein A/G PLUS Agarose (Santa Cruz, CA) at 4°C. The beads were then washed three times, suspended in sample buffer, boiled, and centrifuged, and the supernatants were subjected to immunoblotting against monoclonal anti-Sirt3 or anti-nitrotyrosine, clone1A6, antibodies.

### 2.15. Statistical Analyses

Statistical analyses were performed using GraphPad Prism 5.01. Data are presented as means ± SEM, and significant differences between groups were assessed using one-way analysis of variance (ANOVA) followed by Bonferroni's multiple comparison test or Dunnett's test [47].

## 3. Results

### 3.1. Oxidative Characteristics and ROS Production of Isolated Brain Mitochondria Are Inversely Modified in WT and p66^Shc(−/−)^ Aged Mice

Aiming at understanding how p66^Shc^ participates in the redox homeostasis balance in aging, several biochemical techniques were used to determine different oxidative parameters in purified brain mitochondria from young (3-month-old) and old (24-month-old) WT and p66^Shc−/−^ mice. As shown in Table 1A, there was a significant increment in H_2_O_2_ production by WT mice during aging. This increase in the production rate varied based on the use of malate-glutamate or succinate as the electron transport chain (ETC) substrate, being 33% and 25%, respectively (*p* < 0.05). In turn, p66^Shc−/−^ mice exhibited a significant reduction in total mitochondrial H_2_O_2_ production with both substrates when compared to WT mice (41% and 26%, resp., *p* < 0.05) within the aged mouse groups. Concomitantly, superoxide generation (O_2_^−^) and ROS production rate, measured with MitoSOX and H_2_DCFDA, increased steeply in the WT group during aging (92% and 46%, resp., *p* < 0.05) while in aged KO mice a slight rise was observed in comparison to the young WT control group (38% and 8%, resp., *p* < 0.05) (Table 1B and C).

On the other hand, when the antioxidant detoxification response was analyzed, our results showed that the loss of MnSOD enzymatic activity registered in aging was more pronounced in WT mice than in the aged KO group when contrasted to the 3-month-old WT control (19% and 13%, resp., *p* < 0.05). No changes were observed in catalase activity (Table 1D and E). No differences were detected in any parameters between WT and p66^Shc−/−^ young mouse groups (data not shown).

### 3.2. nNOS Activity and RNS Production Are Altered in Old Transgenic p66^Shc(−/−)^ Mice

**Figure 1:**
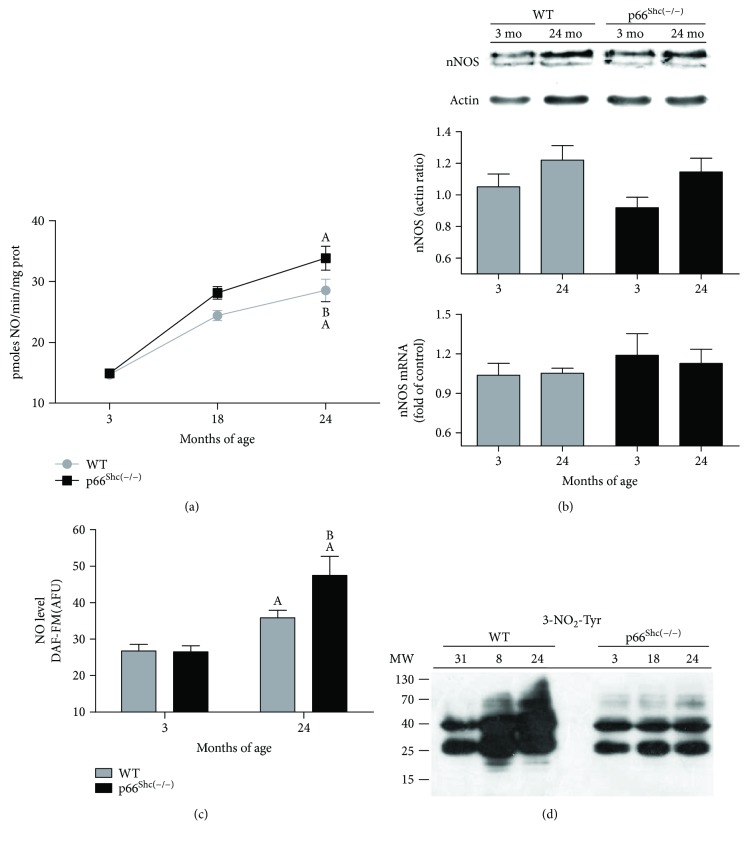
nNOS expression and RNS production in p66^Shc(−/−)^ brain mice during aging. Mice were sacrificed at 3, 18, and 24 months of age. (a) Time course of nNOS activity in mitochondria brain mice measured by 3H-L-citrulline formation (*n* = 6). (b) RT-qPCR and immunoblot of proteins separated using SDS-PAGE from whole brain lysate reveals nNOS mRNA levels and protein expression of mouse brain in WT (grey) and p66^Shc(−/−)^ (black) groups at 3 and 24 months of age. GADPH mRNA levels and actin protein expression were used as housekeeping control and loading control, respectively (*n* > 5, for each experimental group). (c) NO levels were obtained from 50 *μ*g/mL of isolated mitochondria incubated with 0.3 mM L-arginine, 10 *μ*M DAF-FM, and 0.5 *μ*M MitoTracker (per duplicate) at 495 nm (excitation) and 515 nm (emission) in the presence or absence of the NOS inhibitor (3 mM L-NAME) from (*n* ≥ 6). (d) Western blot of nitrated proteins from the different groups; membranes were revealed with anti-3-nitrotyrosine antibodies for each group (*n* ≥ 3). Values represent means ± standard error of the mean (SEM); A represents *p* < 0.05 compared to the 3-month-old group, and B represents *p* < 0.05 between 24-month-old WT and p66^Shc(−/−)^ groups, one-way analysis of variance (ANOVA) and Bonferroni post hoc test.

Considering the low levels of ROS production in p66^Shc(−/−)^ aged mice and its key role in RNS formation during aging, it was interesting to assess how the levels of the nNOS enzyme, and its products and byproducts, were modified in old p66^Shc(−/−)^ mouse brains. The absence of p66^Shc^ did not affect nNOS gene transcription or protein levels in the lifespan of mice.  Figure 1(a) shows a significant increase in nNOS activity of p66^Shc(−/−)^ mice during the latest stage of life compared to WT mice (*p* < 0.05). However, a not significant increase in nNOS content was observed in both groups during aging by Western blot and real-time PCR (Figure 1(b)). In turn, the increase in nNOS activity registered at 24 mo observed in KO mice correlates with the higher NO content measured at the same period (*p* < 0.05) (Figure 1(c)). Interestingly, aged p66^Shc−/−^ mice did not exhibit the same mitochondrial protein nitration profile as aged WT mice did. The latest showed an increment of nitrated proteins compared to the KO group (*p* < 0.05) (Figure 1(d)), consistent with the reduction in H_2_O_2_ and O_2_^−^ production, which is necessary for the reaction with NO to form ONOO^−^.

### 3.3. Altered ROS and RNS in p66^Shc^ KO Mice Modify Brain Mitochondrial Metabolic Parameters in Aging

To understand the effect of RNS and ROS imbalance on the mitochondrial function of aged p66^Shc(−/−)^ mice, isolated and purified brain mitochondria from both groups were used to assess oxygen consumption in ADP stimulation (stage 3) and resting (stage 4) stages. This allowed the determination of the respiratory control ratio (RCR = E3/E4).  Table 2 shows the bioenergetics status measured in brain mitochondria, with malate/glutamate or succinate as oxidizable substrate. Aging decreased the oxygen consumption rate by 25% in stage 3 regardless of the substrate used for the WT group (*p* < 0.05). However, using Mal/Glut as substrate, stage 3 of the respiratory rate was increased by 22% and 68% in aged p66^Shc(−/−)^ mice when compared to the 3-month-old and 24-month-old WT groups, respectively (*p* < 0.05). Meanwhile, this effect decreased by using succinate as substrate with no differences observed between the aged KO and young WT groups and only a 30% increment between the aged KO and the aged WT groups (*p* < 0.05). The respiratory control rates using both Mal/Glut and succinate exhibited an approximate 30% inhibition in aging (*p* < 0.05). In turn, 24-month-old KO mice maintained RCR values similar to those in 3-month-old WT mice (Table 2A). These data suggested an inhibitory effect on the respiratory chain by p66^Shc^ in aged WT mouse brain.

To explain this increase in oxygen consumption rate, the enzymatic activity of the mitochondrial respiratory chain complexes was studied. Results showed that 24-month-old WT mice displayed a higher decrease (48%) in complex I (CI) activity than did aged p66^Shc(−/−)^ mice (34%) when compared to the 3-month-old WT control group (*p* < 0.05) (Table 2). Such decrease in CI activity between the aged groups was noticeable only in the latest stage of life while no differences were observed between the genotypes in previous stages of life (data not shown). Opposite to CI, higher NO concentrations, such as the one observed in p66^Shc(−/−)^ old mice, could be partially responsible for the greater CIV reversible inhibition shown in 24-month-old p66^Shc(−/−)^ mice (31%) in comparison with the decrease observed in WT aging mice (17%) (*p* < 0.05). No differences were observed in CII–III activity profiles during aging between both genotypes (Table 2B).

Table 2C also shows a decrease in brain tissue ATP content for the aged WT group (65%), although such effect was only partially reverted in aged p66^Shc(−/−)^ mice (35%) (*p* < 0.05). This was extensive to other bioenergetics parameters. When we analyzed the NAD^+^/NADH ratio, p66^Shc(−/−)^ mice exhibited a milder decrease (32%) in comparison to the 78% reduction observed in the WT group during aging while the mitochondrial membrane potential (∆Ψ) showed a 14% decrease compared to the 49% decline observed in the same group (*p* < 0.05) (Table 2D and E). Taken together, these results indicate that aged p66^Shc(−/−)^ mice displayed a better mitochondrial function condition compared than aged WT did.

### 3.4. Mitochondrial Content, Ultrastructure, and Morphology Are Modified in Aged p66^Shc(−/−)^ Mouse Brain

**Figure 2:**
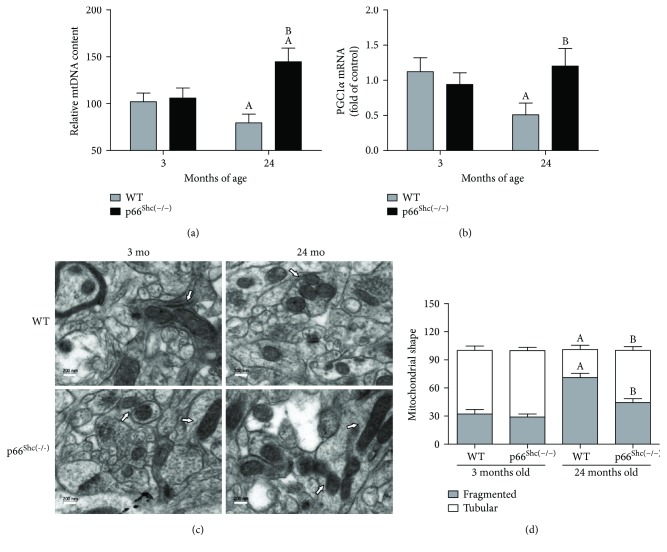
Effects of aging on mitochondrial content, biogenesis, and structure in p66^Shc(−/−)^ mouse brain. (a) Quantification of mtDNA and nuclear DNA by qPCR. (b) The mtDNA/nDNA ratio was calculated from 3- and 24-month WT (grey) and p66^Shc(−/−)^ (black) groups. The mRNA levels of PGC-1*α* were measured in TRIzol-treated brain extracts from all the studied groups; GADPH mRNA was used as standard (*n* = 6). (c) Mitochondrial morphology was evaluated using electron microscopy of fixed brain slices (*n* = 5 for each experimental group) (magnification:×20,000). (d) At least 300 tubular and fragmented mitochondria were counted per arbitrary area. The percentage distribution of tubular and fragmented brain mitochondria was determined in a minimum of 8–10 random fields at ×4400 magnification to ensure a representative area of analysis (*n* = 5). Mitochondria whose length was more than three times their width were considered tubular, while the remaining round mitochondria were considered fragmented. The analysis was performed by two different investigators in a blinded fashion. Values represent the mean ± SEM; A represents *p* < 0.05 compared to the 3-month-old group, B represents *p* < 0.05 between 24-month-old WT and p66^Shc(−/−)^ groups, one-way analysis of variance (ANOVA) and Bonferroni post hoc test.

Based on the improved bioenergetics parameters observed in p66^Shc(−/−)^ mice and the already accepted downregulated mitochondrial biogenesis in aging, it is interesting to study how p66^Shc^ impacts on mitochondrial quantity and structure. The mitochondrial content of a sample can be determined using different methods that provide information about mitochondrial biogenesis and tissue's oxidative capacity. Mitochondrial DNA (mtDNA) content relative to nuclear DNA was determined by real-time qPCR in brain samples of the study groups and shown as a percentage of the 3-month-old WT relative mtDNA content. During aging, mitochondrial content was reduced by 30% in the WT group, while in the p66^Shc(−/−)^ group, a 45% increase was observed (*p* < 0.05) (Figure 2(a)).

In accordance with these results, the PGC-1*α* mRNA expression level declined by 50% in WT mouse brains during aging, whereas in the p66^Shc(−/−)^ group, PGC-1*α* remained stable through their lives (*p* < 0.05) (Figure 2(b)). Electron microscope images with fresh brain slices showed differences in mitochondrial morphology (Figure 2(c)). For both WT and p66^Shc(−/−)^ 3-month-old mice, normal size and volume mitochondria were observed, with predominantly tubular-shaped mitochondria (70% of total measured mitochondria), while the remaining displayed round (fragmented) morphology. However, at the end of their life, the time point of maximal ROS production, 24-month-old WT mouse brain slices were characterized by decreased tubular mitochondria (−44%) and increased round-shaped mitochondria (+120%) (*p* < 0.05). This age effect in mitochondrial morphology was partially mitigated in 24-month-old p66^Shc(−/−)^ mice, to the extent that both types of mitochondrial populations coexisted in this group (55% tubular mitochondria) showing an intermediate phenotype between 3- and 24-month-old WT mice (*p* < 0.05) (Figure 2(d)).

### 3.5. The Absence of p66^Shc^ Is Associated with Changes in Mitochondrial Dynamics in Aging

**Figure 3:**
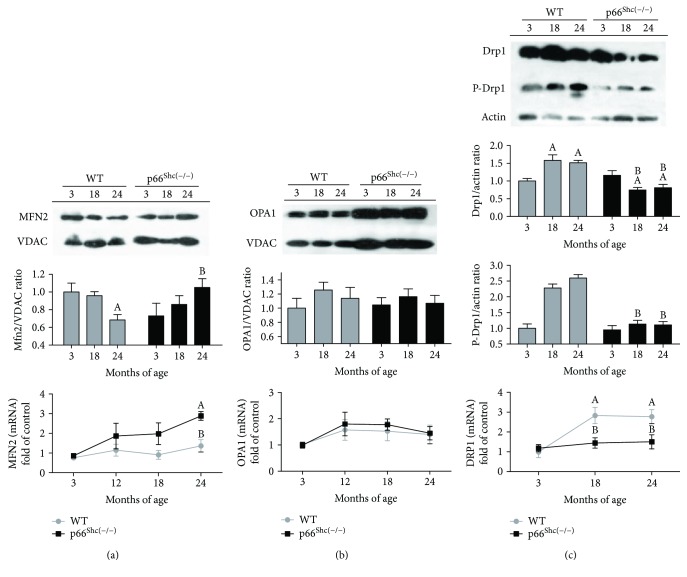
Effects of aging on mitochondrial dynamics in p66^Shc(−/−)^ mouse brain. Immunoblot of proteins separated using SDS-PAGE from purified brain mitochondria or whole brain lysate and RT-qPCR of mRNA from TRIzol-treated brain extracts were performed. The expression of the fusion proteins Mfn2 (a) and Opa1 (b), and the fission protein Drp1 (c) with its (S616) phosphorylated active isoform and mRNA levels of mouse brain in WT (grey) and p66^Shc(−/−)^ (black) groups were observed at 3, 18, and 24 months of age. VDAC protein expression in mitochondrial fraction, actin protein expression in whole brain lysates, and GADPH mRNA levels in RT-qPCR were used as standard, respectively (*n* > 5, for each experimental group). Values represent the mean ± SEM; A represents *p* < 0.05 compared to the 3-month-old group, B represents *p* < 0.05 between 18- or 24-month-old WT and p66^Shc(−/−)^ respective groups, one-way analysis of variance (ANOVA) and Bonferroni post hoc test.

Mitochondrial morphology depends on the interaction among highly dynamic processes, such as the fusion/fission balance. To understand how p66^Shc^ intervenes in mitochondrial dynamics, we analyzed changes in some of the main proteins responsible for the mitochondrial network remodeling.  Figure 3(a) shows that in WT mice, the Mfn2 fusion protein content in the mitochondrial fraction decreased with aging (30%), while p66^Shc(−/−)^ mice exhibited a rise in the levels of this protein (25%) (*p* < 0.05). Additionally, Mfn2 mRNA levels during aging remained steady in the WT genotype while the KO group presented a rising tendency which became significant for the 24-month-old group (3-fold increase of control) (*p* < 0.05) (Figure 3(a)). Unlike Mfn2, no differences were registered either in the Opa1 levels in the mitochondrial fraction of the same tissue or in its messenger expression (Figure 3(b)). To further comprehend the effect of p66 in the mitochondrial network, we also studied a mitochondrial fission dynamin-related protein (Drp1). This protein was downregulated (30%) in KO mice during the latest stages of life, while in the WT group an upregulation of Drp1 was observed during aging (50%) (*p* < 0.05). An increase in p-Drp1 (S616) levels is shown in WT mice throughout their lives (2.5-fold) (*p* < 0.05). However, no changes were detected on p-Drp1 (S616) content in p66^Shc(−/−)^ mouse brain during aging (Figure 3(c)). The pattern observed in Drp1 protein levels throughout the lives of both WT and p66^Shc(−/−)^ mouse groups was similar to that of their gene expression, exhibiting a rise in aged WT mouse brain (3-fold) and remaining stable in the p66^Shc(−/−)^ group, as shown in the same figure (*p* < 0.05).

### 3.6. Sirt3 Activity Is Sensitive to Being Downregulated by Nitration

**Figure 4:**
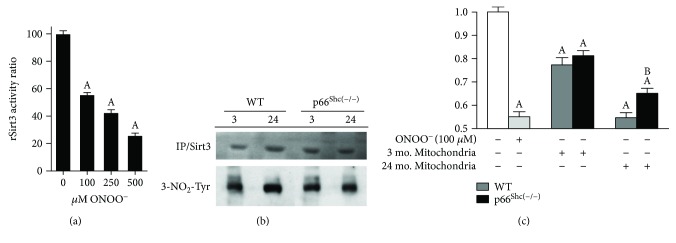
Effect of ONOO^−^ in Sirt3 activity. Recombinant Sirt3 (rSirt3) activity was examined using a fluorometric method. Assays were performed according to the manufacturer's instructions. (a) Recombinant Sirt3 (rSirt3) was incubated with increased concentrations of ONOO^−^ (100, 250, and 500 *μ*M). (b) Immunoprecipitation of Sirt3 was performed from mitochondrial brain lysates incubated with monoclonal anti-Sirt3 antibody and protein A/G PLUS Agarose and then were subjected to immunoblotting against monoclonal anti-Sirt3 or anti-3-nitrotyrosine antibodies. (c) rSirt3 was incubated with 100 *μ*M ONOO^−^ or purified brain mitochondrial lysates from 3- and 24-month WT (grey) and p66^Shc(−/−)^ (black) groups (*n* ≥ 3, for each experimental group). Results were contrasted in pairs between rSirt3 and the two aged groups (a) as well as between both the 24-month-old WT and p66^Shc(−/−)^ groups (b). Values represent means ± SEM; A and B represent *p* < 0.05, one-way analysis of variance (ANOVA) and Bonferroni post hoc test.

To further comprehend how the high levels of RNS, more precisely ONOO^−^, can modulate the deacetylating capacity of mitochondria, we studied the biochemistry of Sirt3, which is the main protein responsible for this process in this organelle. Its biological activity depends on NAD as a cofactor which increased in old p66^Shc(−/−)^ mice (Table 2D). Using the bioinformatics software GPS (Group-Based Prediction System), which allowed us to predict different posttranslational modifications, we analyzed the murine aminoacidic sequence of Sirt3 deacetylase and detected a tyrosine susceptible to be nitrated at position 100. Such position has been reported to be a part of the NAD-binding domain. With the use of a commercial kit, we tested if Sirt3 activity was sensitive to nitration. Incubating a recombinant Sirt3 (rSirt3) with increasing concentrations of ONOO^−^ (100, 250, and 500 *μ*M), we observed a decrease in the enzymatic activity by 47%, 55%, and 70%, respectively (*p* < 0.05) (Figure 4(a)). Furthermore, the 3-nitrotyrosine content of the Sirt3 protein immunoprecipitated from purified mitochondria showed a higher increase on Sirt3 nitration in the WT group compared to the p66^Shc(−/−)^ group during aging. No age- or genotype-related differences were observed in the Sirt3 expression levels measured by immunoprecipitation assay with anti-Sirt3 antibodies (Figure 4(b)). In agreement with these results, rSirt3 activity in the presence of the mitochondrial fraction from aged p66^Shc(−/−)^ mouse brain was higher than that observed when challenged with the aged WT mitochondrial fraction (*p* < 0.05). This inhibitory effect was equivalent to the one obtained with 100 *μ*M ONOO^−^ incubation, while aged p66^Shc(−/−)^ mitochondria with increased NAD/NADH ratio and reduced mitochondrial protein nitration content showed a favorable and less inhibitory environment for rSirt3 activity (33%) (*p* < 0.05) (Figure 4(c)).

## 4. Discussion

Mitochondria play a key role maintaining cellular metabolic homeostasis during aging [48]. In this study, we show that brain mitochondria of p66^Shc(−/−)^ aged mice exhibit a reduced alteration of redox balance and a strong link between reactive nitrogen species and mitochondrial function, morphology, and biogenesis [49]. We also demonstrate that sirtuin 3 lysine deacetylase activity is modulated by its nitration status and is enhanced by the improved NAD^+^/NADH ratio observed in aged p66^Shc(−/−)^ brain mitochondria.

Brain mitochondria of p66^Shc(−/−)^ aged mice exhibit a reduced alteration of redox balance with a decrease both in ROS generation and in its detoxification activity (Table 1), with this balance being the major determinant of lifespan according to Harman's theory of aging. Although this reduction in the antioxidant response is unexpected, the upregulation of this compensatory mechanism might not be necessary considering this low ROS production condition. Young p66^Shc−/−^ mouse brains show normal baseline levels of intracellular oxidative stress compared to wild types, and other groups reported the upregulation of brain p66-Shc gene expression during aging [50]. These findings can explain the reduced age-related oxidative stress levels observed in p66^Shc−/−^ mice. Therefore, p66^Shc^ can be considered a convergent point between both oxidative stress and programmed genetic theories of aging.

Considerable efforts had been made to understand and explain the effects of oxidants (ROS and RNS) on the resultant oxidative-nitrosative stress on lipids, proteins, and DNA and their contribution to the aging process. During the last years, different groups including ours confirmed that NO is a typical mitochondrial modulator. High levels are harmful and end in protein oxidation and nitration, leading to loss of mitochondrial and cell function, while controlled levels can induce stress resistance and mitochondrial biogenesis [51]. Considering that mitochondrial NO metabolism involves regulatory aspects of ROS production, RNS markedly contribute to the mitochondrial modulation of life processes [52]. Indeed, a significant nNOS increase in rat brain during aging has been recently proven to correlate with an increase in mitochondrial protein nitration [8]. Our findings show an intensification in nNOS activity and high levels of NO in p66^Shc(−/−)^ mouse brain during aging (Figure 1). However, the rise in NO production is not followed by protein nitration. A similar effect was previously shown in p66^Shc(−/−)^ mice, which are protected against age-related endothelial dysfunction due to the increment of NO bioavailability without protein nitration [53]. This remarkable effect observed in aged p66^Shc(−/−)^ mice could also be caused by their lower H_2_O_2_ and O_2_^−^ levels, disfavoring the formation of ONOO^−^ by NO breakdown.

Prior evidence has reported a back-and-forth communication flow between changes in ROS/RNS balance and mitochondrial biogenesis, dynamics, and function [10, 54, 55]. This study displays a minor decrease in the respiratory chain complex I (CI) activity with increased oxygen consumption and ATP content in p66^Shc(−/−)^ compared to WT mouse brain during aging, possibly caused by the irreversible nitration of CI at high ONOO^−^ concentrations observed in the last ones (Table 2) [49]. Meanwhile, the inhibition of cytochrome c oxidase (CIV) observed in p66^Shc(−/−)^ brain mitochondria, possibly due to the high levels of steady-state matrix NO concentration, is not enough to counteract this improvement in mitochondrial bioenergetics. Even though NO binds to and reversibly inhibits cytochrome c oxidase-modulating respiration, CI would own the highest level of control in brain mitochondria ETC [56].

Sirt3 is part of the mitochondria-localized sirtuins, modulating acetylation of many proteins that participate in redox regulation [21, 22]. One of the most interesting attributes of Sirt3 is its potential to promote the extension of lifespan [57, 58]. Even though the molecular basis for lifespan extension is not yet clear, the upregulation of Sirt3 expression in long-lived individuals and its physical interaction inside mitochondria with proteins related to the aging process, such as FOXO3a or acetyl-coA synthetase (AceCS2), have been indeed previously studied in literature. The FOXO family transcription factors are human homologs of the daf-16 gene in *Caenorhabditis elegans*, which contributes to the regulation of the nematode lifespan [22]. Additionally, the Sirt3/AceCS2 complex has a role in apoptosis and growth regulation under certain environmental conditions in a specific type of noncancer epithelial cells [59].

Sirt3/PGC-1*α* decreased oxidative stress and conferred resistance to oxidative stress-induced damage by regulating antioxidant defense [60]. In aging, the gradual decline in mitochondrial content results from an age-dependent reduction of the PGC-1*α* level, a key regulator of mitochondrial biogenesis [61, 62]. SIRT3 mediates the reduction of the ROS level by stimulating PGC-1*α* gene expression, and in turn, PGC-1*α* induces SIRT3 expression, creating a positive-feedback loop [27]. Aged p66^Shc(−/−)^ mice show high levels of PGC-1*α* gene expression and increased mitochondrial content compared with their WT littermates (Figure 2). Accordingly, an impaired balance between fission and fusion events may also be related to an age-dependent decline in mitochondrial biogenesis [63].

Mitochondrial morphology depends on the interaction of highly dynamic processes, such as the fusion/fission balance. In aging, given the loss of plasticity of these processes, the maintenance of metabolic homeostasis is compromised. It has been described that ROS-induced mitochondrial depolarization might be responsible for mitochondrial fragmentation [64]. In this work, aged p66^Shc(−/−)^ mouse brain mitochondria present enhanced ∆Ψ and display filament or tubular shape. This is consistent with the observed Mfn2 upregulation, a GTPase embedded in the mitochondria outer membrane directly involved in its fusion, and with the downregulation of Drp1 expression (Figure 3), a GTPase that generates ring-like structures constricting mitochondria in fission events. Phosphorylation of Drp1 at Ser 616 (S616) by several kinases including the cyclin-dependent kinase (CDK) family promotes mitochondrial fission [65]. We also observe high levels of pDrp1-S616 in WT mouse brains during aging. These findings suggest an intimate relationship between mitochondrial energetic status and dynamics. Moreover, regulation of these GTPase proteins by posttranslational modification, such as deacetylation by Sirt3 or nitrosylation/nitration by RNS, is extensively described in literature [15, 28].

Interestingly, the utilization of a GPS algorithm predicts the existence of a tyrosine in position 100 of a Sirt3 murine sequence which, as part of the described NAD^+^-binding domain, is capable of being nitrated [66]. It is known that the decline in NAD^+^ level during aging disrupts nuclear-mitochondrial communication [67]. However, the level of NAD^+^ remains stable in p66^Shc(−/−)^ mice throughout their lives. In accordance to these data, we show here that Sirt3 is sensitive to be nitrated and its activity is protected against age-related nitration in brains of aged p66^Shc(−/−)^ mice (Figure 4). These findings suggest a close relationship between RNS and deacetylation activity in brain mitochondria.

In accordance to the free radical theory of aging, this process is triggered and sustained by the deleterious accumulative effect of oxidative damage caused by mitochondrial respiration-generated ROS. On the other hand, the genetic programmed theory asserts that aging and death are necessary parts of evolution, and the low variability in lifespan within species proves that aging is not only a wear-and-tear process [68–72]. Through its capacity to generate hydrogen peroxide (H_2_O_2_), p66^Shc^ is a clear example of this strong link between oxidative stress and the genetic of aging, and it could be a potential target for anti-aging strategy. The modulation of p66^Shc^ activity could be a candidate for therapeutic intervention for a longer lifespan or higher quality of life.

## 5. Conclusions

In conclusion, results show the pivotal role of p66^Shc^ inactivation in multiple processes related to aging in mouse brains. Through the modification in ROS/RNS production, which in turn regulates Sirt3 activity, the absence of p66^Shc^ improves mitochondrial dynamics and biogenesis. Taken together, these findings can partially explain the observed delay in the aging of p66^Shc(−/−)^ mouse brains. Moreover, the enhancement in mitochondrial homeostasis can be the underlying cause of the improved cognition and motor function observed in p66^Shc^ KO mice during aging [34]. More studies will still be necessary to fully understand this complex and multifactorial process.

## Figures and Tables

**Table 1:** Oxidative parameters in isolated brain mitochondria of wild-type and p66^Shc(−/−)^ aged mice.

	Control (WT 3 mo)	WT 24 mo	p66^Shc(−/−)^ 24 mo
A. H_2_O_2_ production rate (nmoles/min/mg prot)			
1. Malate + glutamate	1.66 ± 0.05	2.22 ± 0.1^a^	0.97 ± 0.03^b^
2. Succinate	1.08 ± 0.05	1.35 ± 0.09^a^	0.8 ± 0.1^b^
B. Superoxide anion level (AFU)	101 ± 8	194 ± 4.5^a^	140 ± 14^b^
C. ROS level (AFU)	76 ± 3	111 ± 8^a^	82 ± 4^a^
D. SOD activity (pmoles/min/mg prot)	2824 ± 309	2305 ± 153^a^	2468 ± 94^a^
E. Catalase activity (units/mg prot)	32 ± 5	29 ± 4	27 ± 3

Note: data are expressed as the mean ± SEM of the different groups (*n* = 6). Results were contrasted in pairs between the 3-month-old WT control group and the two aged groups (a) as well as between the 24-month-old WT and p66^Shc(−/−)^ groups (b). a and b represent *p* < 0.05, one-way analysis of variance (ANOVA) and Bonferroni post hoc test.

**Table 2:** Bioenergetic parameters of isolated brain mitochondria of wild-type and p66^Shc(−/−)^ aged mice.

	Control (WT 3 mo)	WT 24 mo	p66^Shc(−/−)^ 24 mo
A. Oxygen consumption rate			
1. Malate + glutamate			
State 3 (ng at O/min/mg protein)	102 ± 3.6	74 ± 2.4^a^	125 ± 4.6^b^
Respiratory control (RC)	4.3 ± 0.2	3.3 ± 0.1^a^	4.4 ± 0.2
2. Succinate			
State 3 (ng at O/min/mg protein)	116 ± 4.4	84 ± 3.5^a^	109 ± 5^a^
Respiratory control (RC)	4.1 ± 0.1	3.1 ± 0.1^a^	5 ± 0.1^a^
B. Respiratory chain complex activity			
1. Complex I (nmol/min/mg protein)	78.4 ± 4.7	37.5 ± 3.1^a^	51.8 ± 5.2^b^
2. Complex II–III (nmol/min/mg protein)	98.2 ± 9.1	51.1 ± 6.7^a^	48.4 ± 4.1^a^
3. Complex IV (*k*′/min/mg protein)	23 ± 0.75	19.1 ± 0.45^a^	15.8 ± 1^b^
C. ATP synthesis rate (nmol/min/mg protein)	125 ± 1	42 ± 3^a^	81 ± 2^b^
D. NAD^+^/NADH^+^H ratio	5.3 ± 0.7	1.2 ± 0.4^a^	2.7 ± 0.5^b^
E. Mitochondrial potential, ∆Ψ (AFU)	601 ± 15	414 ± 25^a^	520 ± 16^b^

Note: data are expressed as the mean ± SEM of the different groups (*n* = 6). Results were contrasted in pairs between the 3-month-old WT control group and the two aged groups (a) as well as between both the 24-month old WT and p66^Shc(−/−)^ groups (b). a and b represent *p* < 0.05, one-way analysis of variance (ANOVA) and Bonferroni post hoc test. AFU: arbitrary fluorescence units.
